# Is it a complication or a consequence - a new perspective on adverse outcomes in Interventional Radiology

**DOI:** 10.1186/s42155-023-00417-3

**Published:** 2024-01-05

**Authors:** Anna Maria Ierardi, Velio Ascenti, Carolina Lanza, Serena Carriero, Gaetano Amato, Giuseppe Pellegrino, Francesco Giurazza, Pierluca Torcia, Gianpaolo Carrafiello

**Affiliations:** 1 Radiology Department, Fondazione IRCCS Ca’ Granda Ospedale Maggiore Policlinico, Via Francesco Sforza 35, 20122 Milan, Italy; 2 Postgraduate School of Radiology, University of Milan, Milan, Italy; 3 Vascular and Interventional Radiology Department, Cardarelli Hospital, Via A. Cardarelli 9, 80131 Naples, Italy

**Keywords:** Complications, Adverse events, Interventional radiology

## Abstract

The aim of the article is to introduce a new term in post-procedural events related to the procedure itself. All the Societies and Councils report these events as complications and they are divided in mild, moderate and severe or immediate and delayed.

On the other hand the term error is known as the application of a wrong plan, or strategy to achieve a goal.

For the first time, we are trying to introduce the term “consequence”; assuming that the procedure is the only available and the best fit to clinical indication, a consequence should be seen as an expected and unavoidable occurrence of an “adverse event” despite correct technical execution.

## Introduction

According to the Cardiovascular and Interventional Radiology Society of Europe (CIRSE) Classification System for Complications, a complication or adverse event can be defined as any unfavorable and unintended sign (including an abnormal laboratory finding), symptom, or disease temporally associated with the use of a medical treatment or procedure that may or may not be considered related to the medical treatment or procedure [1].

In other words, the complication/adverse event is an unintended injury caused by medical management resulting in measurable disability, prolonged hospital stayed or death [2].

Different is the definition of “error”, defined by the National Academy of Medicine as “the failure of a planned action to be completed as intended or the use of a wrong plan to achieve an aim” [3].

Reason et al. [4] suggested that an error is rarely the result of the action of a single one; instead, there is a complex interaction of events at multiple levels, as represented by the 'Swiss cheese model'. This indicates that there are typically multiple defenses and barriers to error and only if multiple error layers momentarily align, an opportunity for error is produced.

A term that should be introduced to complement the judgment of medical, and specifically of Interventional Radiology (IR)’s practice is that of “consequence”.

The consequence must be defined as something that happened and was predictable, nevertheless could not be avoided (because it was 'inherent' in the procedure), despite the fact that the procedure was performed technically correctly and the procedure was “justified” in terms of indication, to which there are not better alternatives.

Moreover, knowing that a consequence can happen and that it should not be considered a complication, in many cases there are precautions that can be taken before, during or after the procedure to limit clinical manifestations and impact to the patient.

This article aims is to define the difference between “complication” and “consequence”

In the following paragraphs, the most common clinical scenarios will be analyzed, considering different anatomical districts, to identify conditions, which would be better defined as consequences and not complications.

## Before the procedure

The key to a successful IR procedure is a meticulous pre-operative evaluation of the patient and planning of the intervention. Before any IR procedure, the Interventional radiologist (IRs) should review the patient's clinical history and imaging examinations to select and propose the most appropriate procedure verifying indications and eventual absolute or relative contraindications. First of all, medical staff (including IRs) need to examine less invasive and/or more effective alternatives to achieve the set goal. Once it has been established that the interventional procedure is the best choice, according to the CIRSE standards of Practice recommendations, the IRs should discuss with the patient and/or the relatives to explain the procedure, the expected benefits and the potential risks, investigate any allergies, specify which medications would be administered to him and, finally, a written consent should be obtained [5]. During the consultation is fundamental to inform the patient about the most common complications and their frequency. All clinicians, close to the patient during or after the procedure, must be trained to promptly recognize and manage complications.

In most of the IR procedures, the pre-operative phase requires accurate scrutiny of the patient imaging to identify the target, choosing the best and safest approach, in case of failure to be promptly ready to reassess the plan with alternative strategies, and to be aware about the possible complications and how to avoid/manage them.

The IRs should also request and check patient’s pre-procedural lab-tests, including platelet count, blood cell count, hemoglobin levels, and the coagulation profile (prothrombin time (PT), international normalized ratio (INR), activated partial thromboplastin time (aPTT)) [6].

Further targeted testing are conducted based on the patient's clinical profile and the specific procedure being performed. Renal function is examined before procedures requiring the injection of contrast medium, liver function tests establishing Child-Pugh class are necessary prior to liver procedures such as chemoembolization or ablations  [7].

When finally, the patient arrives in the angiographic suite, CIRSE Standards of Practice recommends following a structured periprocedural phase. During the sign-in phase, interventional radiologists are advised to employ a safety checklist to confirm that all the before-mentioned pre-procedural steps have been done correctly. Prior to commencing any treatment, a member of the IR team (e.g., IRs, nurse, radiology technician, resident) should verify that the patient has adhered to fasting requirements, the presence of a functional peripheral venous access and, if necessary, the administration of antibiotic prophylaxis [5].

Pre-operative phase is fundamental in reducing human errors and increasing radiologists’ awareness of the possible obstacles encountered during an interventional radiology procedure and being ready with countermeasures when complications arise.

## Procedures

In the following paragraphs, the most common procedures in which events that are commonly called complications, but could be considered consequences, are reported.

## Lung biopsy and ablation

Imaging-guided transthoracic biopsies or lung ablations are routinely performed in IR departments and are widely considered effective and safe procedures [8]. Albeit in literature the complication rates of these techniques are relatively variable, the most commonly reported adverse events after lung punctures for any occurrence are pneumothorax and pulmonary hemorrhage [9].

### Pneumothorax

A systematic review analyzed 23,104 cases of transthoracic CT-guided lung biopsy, with an incidence of pneumothorax of 25.9%, which required a chest drain insertion for symptomatic patients and for lung compressions exceeding 50% of the total volume in only the 6.9% of cases [10]. The onset of pneumothorax during lung puncture is then a relatively common occurrence owing to the transit of the needle through the pleural space into an aerated tissue - the lung - with the formation of a passage connecting the two tissues. For this reason, pneumothorax is a clinical entity that is only partly dependent on factors that can be modified and controlled, as a large single-cohort study demonstrated by stating that operator experience is only the third major risk factor for pneumothorax occurrence [11].

Risk factors for the development of pneumothorax after a lung puncture include male gender, no history of pulmonary surgery, and pulmonary emphysema; in particular, emphysema was significantly associated with the need for a chest drain insertion, especially in the presence of conspicuous bullae [12]. Factors intrinsic to the target may also influence the pneumothorax rate: deep lesions (>2 cm), small (≤ 2 cm) lesions requiring a longer path to reach the target are factors resulting in a more complex and lengthy procedure [13]. The period in which the needle is inserted in the lung is defined as “dwell time”. Nevertheless, according to the current literature, there is no significant correlation between longer dwell times and an increased incidence of neither pneumothorax nor bleeding, albeit is preferable to accurately plan the procedure and keep the dwell time as low as possible, since patients may start moving or talking after a prolonged time, increasing the risk of needle displacement and of peri-procedural complications [14]. Apart from these non-modifiable factors, there are a series of other situations that seems to relate to a higher rate of pneumothorax that can be modified. Appel et al. evaluated the impact of patient and lesion positioning on pneumothorax rates, concluding that a lower incidence of both pneumothorax and hemoptysis after CT-guided lung biopsy was obtained by positioning the patient in a way that the lesion was below the trachea, reducing the likelihood to damage vascular and respiratory structures near the upper tract airways [15]. This study also concluded that positioning the punctured lung in a dependent position right after the procedure may further reduce the incidence of these complications, benefitting from the “sealing” effect of pressure on the access hole.

Another factor modifiable by the operator is the needle-pleural angle during puncture**.** Needle-pleural angles inferior to 80° have been associated with higher rates of pneumothorax, with the maximum incidence at an angle inferior to 50° [16]. For this reason, the general advice is to try to be as perpendicular to the skin as possible during the procedure. Other maneuvers may reduce the incidence of potential complications [17], including rapid patient rollover, breath-holding techniques, saline or hydrogel tract sealing, and autologous blood patches. The study points out that a regular saline tract sealant could fill the biopsy tract with a water seal, impeding air traveling from the alveoli to the pleural space [18]. Other techniques which significantly reduce the overall rate of pneumothorax and chest tube insertion were the tract plug and blood patch.

Pneumothorax is also reported after percutaneous ablation of primary and metastatic pulmonary lesions [19]. Regardless of the technique utilized (cryotherapy, MWA, multipolar RFA), the use of multiple devices increases the risk of pneumothorax. Also for this procedure, potential patient-related and tumour-related risk factors were described: increased age, male gender, no history of lung surgery and a greater number of tumours ablated. Overall, in about 50% of patients pneumothox does not require a chest tube.

Track ablation is a not recommended maneuver during lung ablations in order to avoid bronchopleural fistula [19].

Therefore, we can affirm that in situations in which percutaneous lung biopsy/ablation represent the best and the only way (ie compared to trans-bronchial) to obtain the diagnosis or to treat the malignancy, there are patients and conditions in which pneumothorax is unavoidable . So a good interventional radiologist must be able to (Table 1):

- ensure the procedure is the best choice of treatment for an individual patient
- recognize the patient's underlying pathology and risk factors
- carry out appropriate and complete pre-procedural planning
- be prepared to admit the patient to the hospital with or without insertion of a chest drain as required
- perform the procedure in the most rapid, safest and technically correct manner
- promptly manage a pneumothorax should one arise (including having a process in place to detect one and make a decision if drainage is required)

**Table 1:** Summary “how to do the best procedure”

	Lung biopsy	Lung ablation	Biliary drainage	Urological drainage	Embolization	Abdominal biopsies, ablations drainages
Indication: best choice for the patient	x	x	x	x	x	x
Hystory: recognize underlying pathology and risk factors	x	x	x	x	x	x
Correct pre-procedural planning	x	x	v	x	x	x
Technique: most rapid, safest and technically correct	x	x	x	x	x	x
Be part of pre and post procedural management	x	x	x	x	x	x
Dialogue with the patient	x	x	x	x	x	x
Be ready to manage complications/consequences	x	x	x	x	x	x

In such a situation as described above where a pneumothorax is inevitable, this should be considered a consequence of a procedure that was deemed the best option for a particular patient regardless of the expected complication.

### Pulmonary hemorrage

Pulmonary hemorrhage is another rather common occurrence during lung ablation or biopsy: in a large cohort featuring 1287 patients undergoing CT-guided biopsy with 18 or 20G needles, a rate of 41% of pulmonary hemorrhage was observed, with only 0.4% cases requiring further treatment or hospitalization [20]. Higher-grade hemorrhages were observed in female patients with characteristics similar to the ones that were predisposed to pneumothorax, such as diffusely emphysematous lung, use of coaxial technique, lesion of small size (< 3 cm), and distant from the pleural surface. The choice of the needle for the biopsy has an important role in the prevention of hemorrhages. In fact, it has been reported that the employment of core needles with throw-length adjustable to tumor size could reduce bleeding risk and even though both tru-cut and end-cut needles have proven their good diagnostic value, end-cut devices are preferred in nodules <10 mm, usually the target more inclined to cause bleedings [21]. Hemorrhage is also rather common during ablation procedures, with a reported rate of 6 – 18% directly proportional to the size of the lesion to ablate, and almost always with a self-limiting history [22]. The most common responsible of bleeding during these procedures are the intercostal arteries, which should be avoided by puncturing the inferior part of the ribs when trying to obtain an intercostal approach.

Haemorrhage requiring blood transfusions or transcatheter embolisation after lung ablation is a rare event (2%) [19].

In conditions in which large vessels are close to the lesions and/or an initial intra-procedural parenchymal bleeding is observed, the most common advice is to continue ablation and to cauterize the path during the removal of the needle/antenna [23].

A good interventional radiologist must be able to (Table 1):

- set up correctly pre-procedural management, perform the most rapid, and technically correct procedure and promptly manage post-procedural phases, as described above.
- ie in case of intra-procedural hemorrhage, continue ablation and consider the possible need to secure the airways (intubation)

## Biliary and urinary drainages

### Biliary

Transhepatic cholangiography (PTC) and percutaneous transhepatic biliary drainage (PTBD) are the most frequent procedures. The most common indications are the stasis of bile flow due to benign or malignant diseases or the accidental lesion of the biliary tree (iatrogenic injury).

Biliary tract disease treatment consists of placing a drain or stent to restore bile flow from the liver to the gastrointestinal (GI) tract.

The most severe complications of PTBD are cholangitis, sepsis, pancreatitis, hemorrhage, fistulae between the bile duct and hepatic artery or portal vein, pseudoaneurysms, bile leaks, and transpleural punctures with risk of pneumothorax or hemothorax [24, 25]. Among them infections and bleeding are the most common.

To reduce the infection rate after PBTD, antibiotic prophylaxis is suggested before the procedure [26].

Cholangitis is more frequent in patients with dilated bile ducts suggesting the presence of cholestasis as a predisposing factor [27].

The transpapillary position of the prosthesis by the internal/external drainage has been associated with a higher incidence of cholangitis, likely because it facilitates migration of bacteria from the bowel into the biliary system. This side effect needs to be balanced against the advantage of restoring the physiological flow of bile to the gut. In addition, from a technical point of view, with internal/external drainages, the distal tip of the drainage is anchored in the foregut to improve stability and reduce the risk of dislocation [27, 28].

Another frequent adverse event is haemobilia, which occours in up to 10% of all cases. Bleeding complications and bilio-vascular fistulas occur in up to 2.5% and 1.5% of cases [25].

A strong correlation between the annual number of PTBD placements per center and complication rates was demonstrated, with more experienced centers having the lowest reported complication rates [29].

IR biliary procedures are indicated in case of failure of Endoscopic retrograde cholangiopancreatography (ERCP) except for rare anatomical or post-surgical conditions [25].

Among the 2 most frequent adverse events reported, we can affirm that in some cases, infective complications are strictly related to the procedure itself and to the patient’s disease.

So a good interventional radiologist must be able to (Table 1):

- set up correctly pre-procedural management, perform the most rapid, safest (i.e. avoiding unuseful transpapillary passages) and technically correct procedure and promptly manage post-procedural phases, as described above.

Therefore in most cases cholangitis is predictable and a good management may be planned in advance. That’s why in several patients, it may be considered a consequence.

### Urinary

The commonest indication for percutaneous nephrostomy (PCN) is urinary tract obstruction [30].

The main post-procedure risk are sepsis and haemorrhage.

It is important to perform a strict observation of the patient in the first 24 hours. After procedure the patient is transferred to the ward and the supervising team (doctors and nurses) has the duty to warm IR if there is any deterioration. A drop in blood pressure may be due to sepsis or haemorrhage and clearly the management will be very different. Laboratory tests and vital parameters need to be monitored [31].

Urosepsis represents one of the most serious complication. Although the incidence is 0.3-4.7%, it can easily lead to infectious shock and with reported 20-40% mortality rate [31, 32].

Factors like positive urine culture, gender and operation time are factors associated to a more frequent urosepsis.

After PCN transient low-grade fever is common. Lee et al reported 100% incidence in 160 patients receiving emergency PCN placement [33]. For the higher complication rates, we can define it as a consequence of the procedure because low-grade fever is due to the introduction of bacteria into the renal area during the procedure. After PCN, infection is common and may also result in pyelonephritis, nephritis and may evolve into septic shock. Urosepsis is a major complication reported in 1 to 3% of all patients and in 7 to 9% of patients with pyonephrosis [30].

So a good interventional radiologist must be able to (Table 1):

- set up correctly pre-procedural management, perform the most rapid, safest (avoiding unuseful passages of guidewires/catheters in the infected urine) and technically correct procedure and promptly manage post-procedural phases, as described above.

As above reported, there are situations in which urosepsis is not avoidable and a good management may be planned in advance. That’s why in several patients, they may be considered consequences

### Biopsies, ablations or drainages of abdominal organs

The most common adverse events associated to abdominal interventional procedures are bleeding, infections, and mechanical complications (e.g. perforation of hollow organs).

Among them, we are going to discuss the perforation of hollow organs, which can be misinterpreted.

In abdominal interventional radiology perforation of hollow organs could be a complication of some procedures such as biopsies, ablation or drainages of collection that are usually localized in the left liver lobe (close to the stomach) and close to the large bowel. The real risk of bowel and gastric perforation in interventional radiology procedures is rare, with reported rates of 0,1 to 2% [34, 35].

Indeed, generally, transection of the small bowel with a small (19 –22 gauge) needle is safe. Contrarily, transection of the colon should be avoided, because of the colonic flora, that in drainage abscess might cause infection of the fluid collection [36].

Appropriate pre-procedural planning and real-time imaging (i.e. US) can reduce the incidence of perforation. Moreover, the employment of intraprocedural techniques such as hydro-dissection or air-dissection might reduce the incidence of these complications.

Therefore, perforation in IR may be an obligated choice, because the operator, aware that it s the only existing path, can choose the trans gastric/duodenal approaches, or to traversing the small bowel. Previously published studies suggested that these approaches are safe and effective [37–40].

Literature report that crossing the gastrointestinal tract with a fine needle is safe and will not result in complications [38].

Moreover, Li et al, in a study on pancreatic biopsies, reported no severe complications after a transgastric approach with an automated 18-20 G cutting needle biopsy gun. The transgastric approach is used in most cases in pancreatic procedures. Patients after the transgastric approach could present mild abdominal pain and elevated serum amylase level that returned to normal within 2 weeks.

A good interventional radiologist must be able to (Table 1):

- set up correctly pre-procedural management, perform the most rapid, safest and technically correct procedure and promptly manage post-procedural phases, as described above.

Assuming that the maneuver is the best and the only available for diagnosis/treatment of the patient, crossing an hollow organ lead to symptoms and signs that me be considered consequences and not complications.

## Embolization

Embolization is a minimally invasive procedure that allows blockage of blood vessels by lodgements of embolic agents (mechanical or liquids). Embolization can be used to stop arterial bleeding, and can also be used to block blood vessels for other reasons, such as to treat tumors, shrink vascular malformations, or re-direct flow [41].

The agent used depends on your medical needs and the type of blood vessel being treated.

Especially in emergency setting, the total time that passes from patient’s injury to the stop of the hemorrhage, is crucial in affecting outcome. Controlling bleeding will only be achieved if coagulopathy is minimized by appropriate blood product support and drug therapy [41].

In any case, the technique and the agent to use is choosen by IRs on the basis of experience and confidence. No guidelines establish what exactly to use and how, but only suggest some good practices.

Non target embolization, massive hemorrhage from intra-procedural vascular rupture or vessel perforation, excessive tissue ischemia resulting in necrosis are the most common complications described after transarterial embolization performed for any kind of indication [41–43].

Outcomes can be variable depending upon embolization target or choice of the embolic agent; other technical considerations include the target organ (kidney presents terminal vascularization: a good embolization must be performed as selectively as possible; mesenteric districts is one of the most noble in body embolization due to the high risk of bowel ischemia if a large area is embolized); thoracic embolization (bronchial, intercostal or lumbar arteries need to be correctly evaluated to avoid the accidental embolization of spinal arteries). These are only some examples to make an idea about the deep knowledge required before facing these procedures.

However there are some situations in which ischemic consequences are unavoidable, for example in massive bleeding (liver, pelvis, spleen ect) in which embolization is a life saving procedure and a proximal embolization is the best choice to contain hemorrhage, restore vital parameters and vicious circle (acidosis, hypothermia, coagulopathy) [44].

In these conditions, ischemic damages are desired consequences and need to be balanced with the value of the life saving.

Spleen injury are worth to be mentioned: splenectomy is associated with an increased risk of developing overwhelming sepsis. IR preserves splenic function and reduces the rate of splenectomy. The benefits of embolization are much higher in severe (grade IV) splenic injuries, where splenic salvage is seen in 84–94% of patients treated with embolization [41].

Techniques of embolization include stent graft placement, proximal and distal embolization. Complications of embolization occur in up to 15% of patients and include recurrent hemorrhage and abscess formation. However, the incidence of infection is lower than with splenectomy.

A good interventional radiologist must be able to (Table 1):

- set up correctly pre-procedural management, perform the most rapid, and technically correct procedure and promptly manage post-procedural phases, as described above.
- ie using mechanical agents in proximal embolization and not liquids that may penetrate deeper in small vessels, giving necrosis of muscles, pancreas, bladder, ect depending on the district involved
- Situations, like the above mentioned are conditions in which ischemia/infarct are expected and a good management makes it a consequence of a good IR practice.

### Post embolization and post ablation syndromes

Post-embolization syndrome (PES) and post-ablation syndrome (PAS) are common phenomenon in the field of interventional radiology and considered as minor complications characterized by the onset of flu-like symptoms within the first 24–48h after the procedure.

PES refers to a constellation of symptoms that arise after embolization procedures, particularly arterial embolization techniques used to treat various medical conditions such as tumors, vascular malformations, and uterine fibroids. PES is generally considered a self-limiting and benign condition, but its clinical presentation can lead to significant discomfort and may warrant appropriate management strategies.

The clinical presentation of PES can vary among individuals and is influenced by factors such as the site of embolization, the extent of tissue ischemia, and the patient's overall health status. Common symptoms associated with PES include pain, fever, fatigue, malaise, myalgia, nausea and vomiting; despite not common, some patients may experience additional manifestations such as headache, sweating, dizziness, and changes in appetite [45].

The exact aetiology of PES remains unclear; however, it is thought that the inflammatory response and Ischemia-Reperfusion Injury (IRI) play an active role in the pathogenesis of this condition. The interruption of blood flow during embolization procedures leads to tissue ischemia and cellular damage, which trigger a cascade of events with the release of pro-inflammatory cytokines and chemokines and the subsequent recruiting of immune cells, such as neutrophils and macrophages, meanwhile the restoration of oxygen and nutrients to previous ischemic tissues causes the production of reactive oxygen species (ROS), amplification of inflammatory pathways, and release of damage-associated molecular patterns (DAMPs) [46, 47].

PES manifestations are usually mild, however, its impact on patients’ Quality of Life (QOL) and length of stay (LOS) is a significant issue. The management of post-embolization syndrome (PES) primarily focuses on symptomatic relief and supportive care. Several strategies have been proposed to prevent or at least reduce patients’ discomfort. Some authors suggested the use of dexamethasone to reduce the clinical impact of PES, nonsteroidal anti-inflammatory drugs (NSAIDs) or opioids may be prescribed to alleviate pain and antiemetic medications can be administered to manage nausea and vomiting [47, 48].

Similarly, post-ablation syndrome (PAS) is described as complication of thermo-ablation techniques (radiofrequency, microwaves and cryoablation) characterized by fever, malaise, myalgia, nausea, vomiting and pain localized at the ablation site [49]. Despite the underlying mechanism is still not clear, the pathophysiology of PAS shares a similar pathway with PES, it is suggested that thermal injury and necrosis trigger the release of pro-inflammatory cytokines and activation of immune cells resulting in systemic inflammatory response causing pain, fever and malaise. The prevalence of PAS following thermo-ablation procedures varies in the literature. Studies have reported incidence rates ranging from 10% to 50%, depending on factors such as the type and extent of ablation, site and patient characteristics. In a similar fashion to PES, NSAIDs or opioids are generally used to treat pain [50, 51].

The high incidence of these conditions makes them highly predictable after embolization or ablation treatments. The suggestion of considering them as consequences instead of complications comes from the need that clinicians and radiologists should expect them as the natural post-operative course of these procedures. Besides, considering PES as an expected consequence rather than a complication is not new. Basile et al. [52] already proposed that PES is a foreseen outcome after TACE, hence it should not be addressed as complications anymore.

A good interventional radiologist must be able to (Table 1):

- set up correctly pre-procedural management, perform the fastest, and technically correct procedure and promptly manage post-procedural phases, as described above.
- i.e., a good strategy to prevent PAS/PES is to carry out pre-medication with dexamethasone and prompt post –procedural management of symptoms

## Discussion

An adverse event in interventional radiology can manifest itself in various forms: there are medical errors, which are established due to erroneous pre- or intra-operative assessment, and there are complications, which by definition are deviations from the regular post-therapeutic course, whether symptomatic or asymptomatic [2, 53]. We believe there is room for the introduction of a new definition, not currently found in the medical literature: consequence. Consequence can be defined as the *expected* and *unavoidable* occurrence of an "adverse event" despite correct technical execution.

A clarifying example might be the occurrence of a subtle pneumothorax after passing through an emphysematous lung in a percutaneous procedure or a post-ablation syndrome that manifests with fever the day after percutaneous hepatic thermo-ablation.

Assuming that the maneuvers represent the best and the only available for diagnosis/treatment, they cannot be considered complications, as generally reported.

These conditions are actually predictable consequences.

Being able interpret a consequence correctly can help to prevent it from turning into a complication. Proper management of such consequences begins with awareness of expecting and implementing all possible strategies to contain the adverse event before, during, and after the procedure.

Also patients should be aware about consequences and complications related to the procedure they are going to underwent. At the moment of the informed consent acquisition or even before that, the dialogue between the patient and the IRs is an important step that has a double purpose: first of all inform the patient about what are the consequences related to the procedure and how they can be managed when they arise, making him aware of what these may entail in the post-procedural course, and then optimize the relationship with the doctor based on the trust and aimed to better accept also the complications. About that it is worth to remember the importance to spend time with the patient (organizing visits, being available to satisfy any requests, and being ready to remain with him in case he needs post procedural assistance). It’s implied that all the staff should be trained in that direction (nurses, technicians, residents).

In our paper, an important concept is coming out: IR procedures are often the only manner to get diagnosis and in some conditions the mainstay of therapy. Our specialty is moving from the delivery of a procedure towards taking care for a patient’s condition with the IRs part or direct responsable for the patient’s outcomes. Those concepts were already treated in clinical practice manual published by CIRSE (5); aspects ranging from facilities, to the relevance of inpatienst and outpations IR consultations, from preprocedural planning, check list and post procedural care of the patient were reported (5).

We would take the opportunity to highlight the role of the IRs from the presentation of the patient, proposals for diagnosis and treatments up to taking care in the post-procedural and follow up period.

Therefore, IR’s responsibility is to coordinate and inform all healthcare professionals involved in patient care in a cross-cutting way, to minimize the risk associated with such consequences but also be part personally as real responsible of the patient.

In fact, as we know, it often happens in IR that patients who need imaging-guided procedures are referred by doctors who follow the patient before and after the procedure. Contrary to surgery, which has historically emphasized total and complete ownership of patient care, IR participation is usually hindered by a lack of continuity [54]. One of the most important need arising from the concepts described above is that IRs position should be pushed as specialist who taking care of the patient underwent to our procedures.

## Conclusions

Systematic use of a more appropriate terminology could have positive influence in patient care and management. Educating and informing clinicians about the expected consequences of the procedure is the responsibility of the IR who is familiar with these conditions and could improve the management of the patient.

## Abbreviations

CIRSE: Cardiovascular and Interventional Radiology Society of Europe
IR: Interventional Radiology
IRs: Interventional radiologist
PT: prothrombin time
INR: international normalized ratio
aPTT: activated partial thromboplastin time
CT: Computed Tomography
MWA: microwave ablation
RFA: radiofrequency ablation
G: gauge
PTC: Transhepatic cholangiography
PTBD: percutaneous transhepatic biliary drainage
GI: gastrointestinal
ERCP: Endoscopic retrograde cholangiopancreatography
PCN: percutaneous nephrostomy
PES: Post-embolization syndrome
PAS: post-ablation syndrome
ROS: reactive oxygen species
DAMPs: damage-associated molecular patterns
QOL: quality of life
LOS: length of stay
NSAIDs: Nonsteroidal anti-inflammatory drugs

## References

[1] Filippiadis DK, Binkert C, Pellerin O, Hoffmann RT, Krajina A, Pereira PL (2017). Cirse quality assurance document and standards for classification of complications: the cirse classification system. Cardiovasc Intervent Radiol.

[2] Higgins MCSS, Herpy JP (2021). Medical error, adverse events, and complications in interventional radiology: liability or opportunity?. Radiology.

[3] Kohn L.T., Corrigan J.M., Donaldson M.S., Institute of Medicine (US) Committee on Quality of Health Care in America (2000). To Err Is Human: Building a Safer Health System.

[4] Reason J (2000). Human error: models and management. BMJ.

[5] Mahnken AH, Boullosa Seoane E, Cannavale A, de Haan MW, Dezman R, Kloeckner R, O’Sullivan G, Ryan A, Tsoumakidou G (2021). CIRSE Clinical Practice Manual. Cardiovasc Intervent Radiol.

[6] Hadi M, Walker C, Desborough M, Basile A, Tsetis D, Hunt B, Müller-Hüllsbeck S, Rand T, van Delden O, Uberoi R (2021). CIRSE standards of practice on peri-operative anticoagulation management during interventional radiology procedures. Cardiovasc Intervent Radiol.

[7] Lucatelli P, Burrel M, Guiu B, de Rubeis G, van Delden O, Helmberger T (2021). CIRSE standards of practice on hepatic transarterial chemoembolisation. Cardiovasc Intervent Radiol.

[8] Azour L, Liu S, Washer SL, Moore WH (2021). Percutaneous transthoracic lung biopsy: optimizing yield and mitigating risk. J Comput Assist Tomogr.

[9] Sabatino V, Russo U, D’Amuri F, Bevilacqua A, Pagnini F, Milanese G, Gentili F, Nizzoli R, Tiseo M, Pedrazzi G, De Filippo M (2021). Pneumothorax and pulmonary hemorrhage after CT-guided lung biopsy: incidence, clinical significance and correlation. Radiol Med.

[10] Huo YR, Chan MV, Habib A-R, Lui I, Ridley L (2020). Pneumothorax rates in CT-Guided lung biopsies: a comprehensive systematic review and meta-analysis of risk factors. Br J Radiol.

[11] Yeow K-M, Su I-H, Pan K-T, Tsay P-K, Lui K-W, Cheung Y-C, Chou AS-B (2004). Risk factors of pneumothorax and bleeding. Chest.

[12] Hallifax R, Janssen JP (2019). Pneumothorax—time for new guidelines?. Semin Respir Crit Care Med.

[13] Heerink WJ, de Bock GH, de Jonge GJ, Groen HJM, Vliegenthart R, Oudkerk M (2017). Complication rates of CT-guided transthoracic lung biopsy: meta-analysis. Eur Radiol.

[14] Ko JP, Shepard J-AO, Drucker EA, Aquino SL, Sharma A, Sabloff B, Halpern E, McLoud TC (2001). Factors influencing pneumothorax rate at lung biopsy: are dwell time and angle of pleural puncture contributing factors?. Radiology.

[15] Appel E, Dommaraju S, Camacho A, Nakhaei M, Siewert B, Ahmed M, Brook A, Brook OR (2020). Dependent lesion positioning at CT-guided lung biopsy to reduce risk of pneumothorax. Eur Radiol.

[16] Maalouf N, Abou Mrad M, Lavric D, Vasileva L, Mahnken AH, Apitzsch J (2023). Safe zone to avoid pneumothorax in a CT-guided lung biopsy. J Clin Med.

[17] Huo YR, Chan MV, Habib A-R, Lui I, Ridley L (2019). Post-Biopsy manoeuvres to reduce pneumothorax incidence in CT-guided transthoracic lung biopsies: a systematic review and meta-analysis. Cardiovasc Intervent Radiol.

[18] Li Y, Du Y, Luo TY, Yang HF, Yu JH, Xu XX, Zheng HJ, Li B (2015). Usefulness of normal saline for sealing the needle track after CT-guided lung biopsy. Clin Radiol.

[19] Venturini M, Cariati M, Marra P, Masala S, Pereira PL, Carrafiello G (2020). CIRSE standards of practice on thermal ablation of primary and secondary lung tumours. Cardiovasc Intervent Radiol.

[20] Tai R, Dunne RM, Trotman-Dickenson B, Jacobson FL, Madan R, Kumamaru KK, Hunsaker AR (2016). Frequency and severity of pulmonary hemorrhage in patients undergoing percutaneous CT-guided transthoracic lung biopsy: single-institution experience of 1175 cases. Radiology.

[21] Echevarria-Uraga JJ, del Cura-Allende G, Armendariz-Tellitu K, Berastegi-Santamaria C, Egurrola-Izquierdo M, Anton-Ladislao A (2022). Complications and diagnostic accuracy of CT-guided 18G tru-cut versus end-cut percutaneous core needle biopsy of solitary solid lung nodules. Diagn Interv Radiol.

[22] Alzubaidi SJ, Liou H, Saini G, Segaran N, Scott Kriegshauser J, Naidu SG, Patel IJ, Oklu R (2021). Percutaneous image-guided ablation of lung tumors. J Clin Med.

[23] Carrafiello G, Mangini M, Fontana F, Massa A, Ierardi AM, Cotta E, Piacentino F, Cardim LN, Pellegrino C, Fugazzola C (2012). Complications of microwave and radiofrequency lung ablation: personal experience and review of the literature. Radiol Med.

[24] Turan AS, Jenniskens S, Martens JM, Rutten MJCM, Yo LSF, van Strijen MJL, Drenth JPH, Siersema PD, van Geenen EJM (2022). Complications of percutaneous transhepatic cholangiography and biliary drainage, a multicenter observational study. Abdom Radiol (NY).

[25] Das M, van der Leij C, Katoh M, Benten D, Hendriks BMF, Hatzidakis A (2021). CIRSE standards of practice on percutaneous transhepatic cholangiography, biliary drainage and stenting. Cardiovasc Intervent Radiol.

[26] D. Bhattacharjee, T. Sheth, A. Adiamah, D. Gomez. Adherence to antibiotic prophylaxis for percutaneous transhepatic cholangiography: a single-centre experience. Cureus. 2020;12. 10.7759/CUREUS.7989. 10.7759/cureus.7989 PMC7274263 32523844

[27] Pedersoli F, Schröder A, Zimmermann M, Schulze-Hagen M, Keil S, Ulmer TF, Neumann UP, Kuhl CK, Bruners P, Isfort P (2021). Percutaneous transhepatic biliary drainage (PTBD) in patients with dilated vs. nondilated bile ducts: technical considerations and complications. Eur Radiol.

[28] Xu C, Huang X-E, Wang S-X, Lv P-H, Sun L, Wang F-A (2015). Comparison of infection between internal-external and external percutaneous transhepatic biliary drainage in treating patients with malignant obstructive jaundice. Asian Pac J Cancer Prev.

[29] Rees J, Mytton J, Evison F, Mangat KS, Patel P, Trudgill N (2020). The outcomes of biliary drainage by percutaneous transhepatic cholangiography for the palliation of malignant biliary obstruction in England between 2001 and 2014: a retrospective cohort study. BMJ Open.

[30] Ramchandani P, Cardella JF, Grassi CJ, Roberts AC, Sacks D, Schwartzberg MS, Lewis CA (2001). Quality improvement guidelines for percutaneous nephrostomy. J Vasc Interv Radiol.

[31] Pabon-Ramos WM, Dariushnia SR, Walker TG, d'Othee BJ, Ganguli S, Midia M, Siddiqi N, Kalva SP, Nikolic B (2016). Quality improvement guidelines for percutaneous nephrostomy. J Vasc Interv Radiol.

[32] Wu H, Fu Y, He Q, Bao J (2023). Risk factors for urosepsis after percutaneous nephrolithotomy: a meta-analysis. Asian J Surg.

[33] Lee WJ, Patel U, Patel S, Pillari GP (1994). Emergency percutaneous nephrostomy: results and complications. J Vasc Interv Radiol.

[34] Carrafiello G, Laganà D, Ianniello A, Dionigi G, Novario R, Recaldini C, Mangini M, Cuffari S, Fugazzola C (2007). Post-radiofrequency ablation syndrome after percutaneous radiofrequency of abdominal tumours: one centre experience and review of published works. Australas Radiol.

[35] De Bazelaire C, Coffin A, Cohen S, Scemama A, De Kerviler E (2014). Biopsies in oncology. Diagn Interv Imaging.

[36] Gervais DA, Brown SD, Connolly SA, Brec SL, Harisinghani MG, Mueller PR (2004). Percutaneous imaging-guided abdominal and pelvic abscess drainage in children. Radiographics.

[37] Sofocleous CT, Schubert J, Brown KT, Brody LA, Covey AM, Getrajdman GI (2004). CT-guided transvenous or transcaval needle biopsy of pancreatic and peripancreatic lesions. J Vasc Interv Radiol.

[38] Brandt KR, Charboneau JW, Stephens DH, Welch TJ, Goellner JR (1993). CT- and US-guided biopsy of the pancreas. Radiology.

[39] Xu K, Zhou L, Liang B, Niu L, Zheng X, Xu J, Yang D, Tan D, Xu K (2012). Safety and accuracy of percutaneous core needle biopsy in examining pancreatic neoplasms. Pancreas.

[40] Stella SF, Van Borsel M, Markose G, Nair SB (2019). Image-guided percutaneous biopsy for pancreatic lesions: 10-year experience in a tertiary cancer center. Can Assoc Radiol J.

[41] Chakraverty S, Flood K, Kessel D, McPherson S, Nicholson T, Ray CE, Robertson I, van Delden OM (2012). CIRSE guidelines: quality improvement guidelines for endovascular treatment of traumatic hemorrhage. Cardiovasc Intervent Radiol.

[42] Rand T, Patel R, Magerle W, Uberoi R (2020). CIRSE standards of practice on gynaecological and obstetric haemorrhage. CVIR Endovasc.

[43] Kettenbach J, Ittrich H, Gaubert JY, Gebauer B, Vos JA (2022). CIRSE standards of practice on bronchial artery embolisation. Cardiovasc Intervent Radiol.

[44] Gerecht R (2014). The lethal triad. Hypothermia, acidosis & coagulopathy create a deadly cycle for trauma patients. JEMS.

[45] Leung DA, Goin JE, Sickles C, Raskay BJ, Soulen MC (2001). Determinants of postembolization syndrome after hepatic chemoembolization. J Vasc Int Radiol.

[46] Eltzschig HK, Eckle T (2011). Ischemia and reperfusion—from mechanism to translation. Nat Med.

[47] Blackburn H, West S (2016). Management of postembolization syndrome following hepatic transarterial chemoembolization for primary or metastatic liver cancer. Cancer Nurs.

[48] Yang H, Seon J, Sung PS, Oh JS, Lee HL, Jang B, Chun HJ, Jang JW, Bae SH, Choi JY, Yoon SK (2017). Dexamethasone prophylaxis to alleviate postembolization syndrome after transarterial chemoembolization for hepatocellular carcinoma: a randomized, double-blinded, placebo-controlled study. J Vasc Int Radiol.

[49] Livraghi T, Solbiati L, Meloni MF, Gazelle GS, Halpern EF, Goldberg SN (2003). Treatment of focal liver tumors with percutaneous radio-frequency ablation: complications encountered in a multicenter study. Radiology.

[50] Wah TM, Arellano RS, Gervais DA, Saltalamacchia CA, Martino J, Halpern EF, Maher M, Mueller PR (2005). Image-guided percutaneous radiofrequency ablation and incidence of post-radiofrequency ablation syndrome: prospective survey. Radiology.

[51] Kawabata T, Hiraki T, Iguchi T, Matsui Y, Uka M, Masaoka Y, Komaki T, Sakurai J, Gobara H, Araki M, Nasu Y, Kanazawa S (2020). Post-ablation syndrome after percutaneous cryoablation of small renal tumors: A prospective study of incidence, severity, duration, and effect on lifestyle. Eur J Radiol.

[52] Basile A, Carrafiello G, Ierardi AM, Tsetis D, Brountzos E (2012). Quality-improvement guidelines for hepatic transarterial chemoembolization. Cardiovasc Intervent Radiol.

[53] Dindo D, Clavien P-A (2008). What is a surgical complication?. World J Surg.

[54] Mafeld S, Oreopoulos G, Musing ELS, Chan T, Jaberi A, Rajan D (2020). Sources of error in interventional radiology: how, why, and when. Can Assoc Radiol J.

